# Related Factors and Outcome of Spinal Cord Stimulation Electrode Deviation in Disorders of Consciousness

**DOI:** 10.3389/fneur.2022.947464

**Published:** 2022-07-04

**Authors:** Qiheng He, Bin Han, Xiaoyu Xia, Yuanyuan Dang, Xueling Chen, Jianghong He, Yi Yang

**Affiliations:** ^1^Department of Neurosurgery, Beijing Tiantan Hospital, Capital Medical University, Beijing, China; ^2^Department of Neurosurgery, China National Clinical Research Center for Neurological Diseases, Beijing, China; ^3^Department of Neurosurgery, Zhongshan Hospital of Traditional Chinese Medicine, Guangdong, China; ^4^Department of Neurosurgery, PLA General Hospital, Beijing, China; ^5^Department of Neurosurgery, Chinese Institute for Brain Research, Beijing, China; ^6^Department of Neurosurgery, Beijing Institute of Brain Disorders, Beijing, China

**Keywords:** disorders of consciousness, spinal cord stimulation, electrode accuracy, outcome, deviation

## Abstract

**Background and Purpose:**

Spinal cord stimulation (SCS) has been reported to be a promising neuromodulation method for patients with disorders of consciousness (DOC). Our previous studies found that clinical characteristics of patients and SCS stimulation parameters could affect the therapeutic effects of SCS, while surgical-related factors remain unknown. Through the improvement of surgical procedures, most of the SCS electrodes are implanted in the middle, while a small number of electrodes have still deviated.

**Methods:**

A total of 137 patients received SCS treatment in our institutions from 1 January 2010 to 31 December 2020. Among them, 27 patients were found with electrode deviation and met the inclusion criteria. Patients were grouped according to whether the electrode deviation angle (EDA) is >30°, respectively. Clinical characteristics of patients and SCS stimulation parameters were compared. Potential related factors and outcomes were evaluated by Chi-square test or two-way repeated measures analysis.

**Results:**

Twenty seven patients receiving cervical SCS treatment were found to have electrode deviation postoperatively. Among them, 12 patients were classified into the more deviation group. No significant difference was found among age, sex, pathogeny, course of DOC, C2–C5 distance, spinal cord to spinal canal ratio at C2 level, and preoperative JFK Coma Recovery Scale-Revised (CRS-R) scores. We found that the electrode direction significantly deviated to the contralateral side in the lateral decubitus position (*P* = 0.025). The maximum tolerant stimulation intensity in the less deviation group (1.70 ± 0.41) was significantly higher than that in the more deviation group (1.25 ± 0.34) (*P* = 0.006). Under the strongest stimulation, less unilateral limb tremor (*P* = 0.049) and paroxysmal sympathetic hyperactivity (PSH) episodes (*P* = 0.030) were found. EDA had a significant effect on postoperative CRS-R in patients, and patients in the less deviation group had significantly higher postoperative CRS-R (*P* < 0.01). There was also an interaction effect between EDA and postoperative time. With the prolonged postoperative time, the CRS-R improvement rate of patients with different EDA was different, and the CRS-R improved faster in patients with less EDA (*P* < 0.05).

**Conclusions:**

Electrode deviation will affect the outcome of patients receiving cervical SCS treatment. The intraoperative surgical position is associated with postoperative electrode deviation direction. The reduction of EDA under 30° can increase maximum tolerant stimulation intensity, reduce complications, and further improve patients' outcomes.

## Introduction

Spinal cord stimulation (SCS) is a therapeutic technology that involves the implantation of electrodes at the midline of the posterior epidural space and delivers electric stimulation to the spinal cord (1, 2). SCS is traditionally used to treat chronic pain syndromes. Since the first report by Kanno in the 1980s, cumulative evidence has proved the efficacy of SCS treatment in patients with disorders of consciousness (DOC), especially for patients in the minimally conscious state (MCS) (3–6). In recent years, the mechanisms of SCS in treating patients with DOC have been reported. The direct stimulation of C2–C4 on the dorsal column could significantly increase cerebral blood flow, promote cortical neuroplasticity, and improve functional activity in certain brain circuits (7–11). As for surgical procedures, a C5 vertebra laminotomy is made and the electrode array is delivered up to the target zone at the midline of the C2–C5 level (12), and the electrode position may affect prognosis and patient tolerance. We preliminary reported stimulation parameters that could affect the therapeutic effects of SCS, such as inter-stimulus interval and frequency (5, 13), and summarized clinical characteristics on patients' outcomes (14). Considering that the mechanism of SCS to treat disturbance of consciousness possibly underlying in the direct stimulation of the dorsal column, and since we observed that patients with electrodes closer to the midline seem to have a better prognosis, we proposed that surgical factors such as electrode deviation may affect the outcomes of patients. However, factors related to surgical procedures and electrode deviation on the prognosis of DOC patients are still unclear.

The aim of this study is to investigate whether electrode deviation will affect the outcome of patients receiving cervical SCS treatment and its related factors. We retrospectively enrolled patients with DOC who underwent SCS treatment in our department and analyzed the related influencing factors, therapeutic effects, and complications on electrode deviation angle (EDA).

## Materials and Methods

### Patients

This multi-center retrospective study enrolled patients receiving SCS treatment in the Department of Neurosurgery, Beijing Tiantan Hospital, Capital Medical University, and Peking University International Hospital from 1 January 2010 to 31 December 2020. The inclusion criteria were: 1) diagnosed as DOC according to the recommendations of the Multi-Society Task Force on the Persistent Vegetative State (15); 2) DOC that lasted for at least 3 months; 3) postoperative CT scan was performed. The exclusion criteria were: 1) significant improvement or deterioration of consciousness within 4 weeks; 2) the level of consciousness reached emerged MCS; 3) severe cervical spinal cord or canal deformity or injury; 4) no postoperative electrode deviation (EDA <5); 5) no postoperative measurement available. Considering the patients could not understand and legally consent to the treatment and enrollment, their legal representatives and family members were given the written formed consent. The study was approved by the ethics committee of Beijing Tiantan Hospital, Capital Medical University (KYSQ 2020 - 387 - 01).

### Data Collection

Data were extracted from the medical records of enrolled patients from 1 January 2010 to 31 December 2020. The baseline variables include sex, age, course, pathogeny, and preoperative evaluation (preoperative JFK Coma Recovery Scale-Revised (CRS-R) (16), cervical spinal cord to spinal canal ratio, etc.).

**Figure 1:**
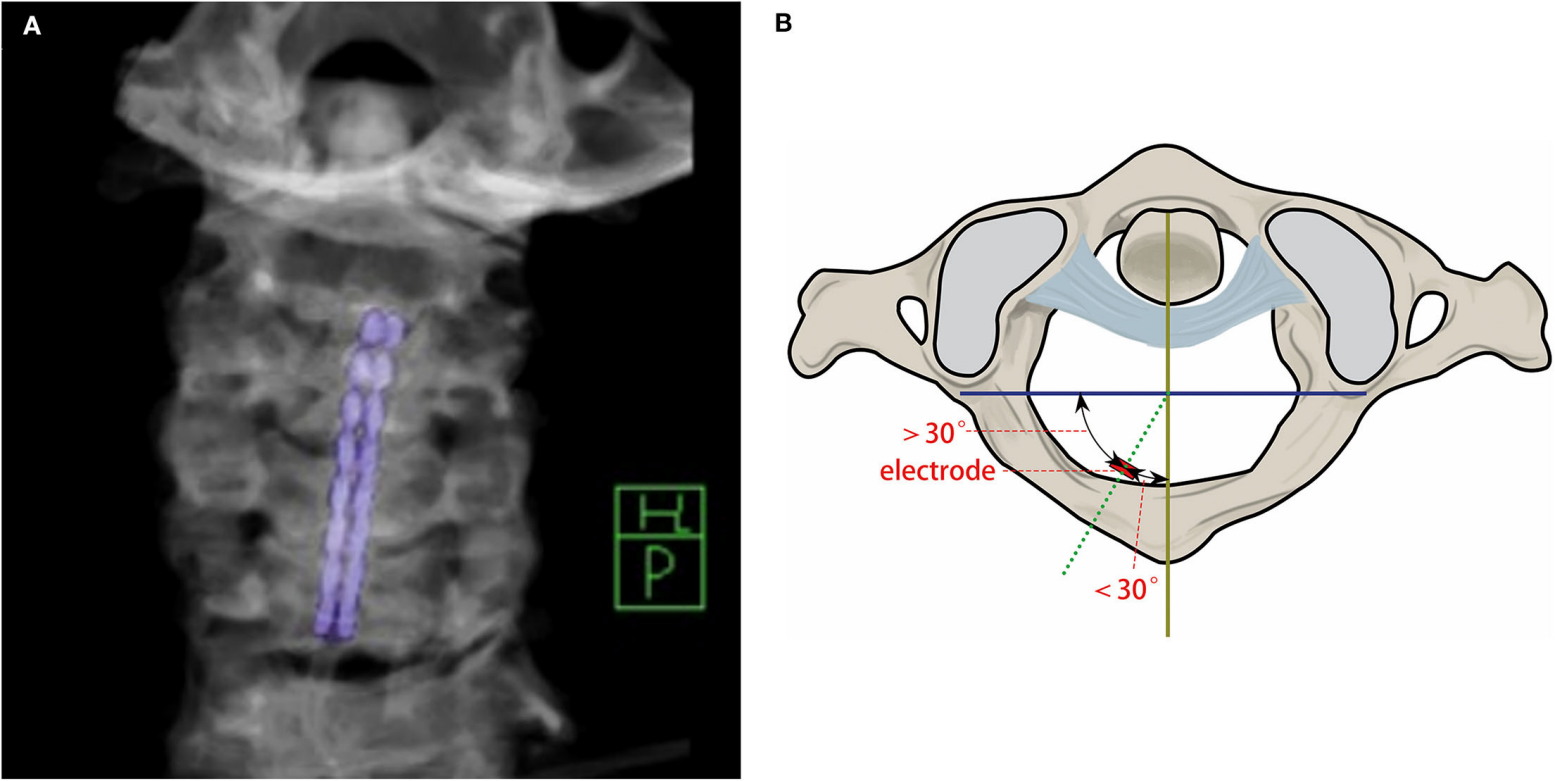
Representative image of electrode deviation. **(A)** VRT reconstruction image using CT scan of the electrode and the vertical midline of the spinal canal. **(B)** Schematic diagram of electrode offset angle.

We also collected the postoperative deviation angle of the 1st, 2nd, and 3rd electrodes of the paddle lead and therapeutic effects (postoperative stimulation parameters, postoperative CRS-R, paroxysmal sympathetic hyperactivity (PSH) episodes, etc.). MRI was performed preoperatively and the C2 cervical spinal cord segment was selected using the PACS system to measure the C2–C5 distance and cervical spinal cord to spinal canal ratio at the C2 level. CT was performed within 3 days postoperatively. Three-dimensional volume rendering technique (VRT) reconstruction of the cervical vertebra was performed and the planes where the 1st, 2nd, and 3rd electrodes are located were selected, respectively. The line connecting the midpoint of the lamina and the spinous process is defined as the vertical axis, and the vertical line passing through the midpoint of the vertical axis is the horizontal axis. The intersection point is set as the origin, and the angle between the line connecting the electrode and the origin and the vertical axis using PACS was measured (Figure 1). Patients with C2 axial electrode deviation angle (EDA) ≤ 30° were classified as the less deviation group, and patients with deviation angle >30° were classified as the more deviation group.

### Surgical Procedure

**Figure 2:**
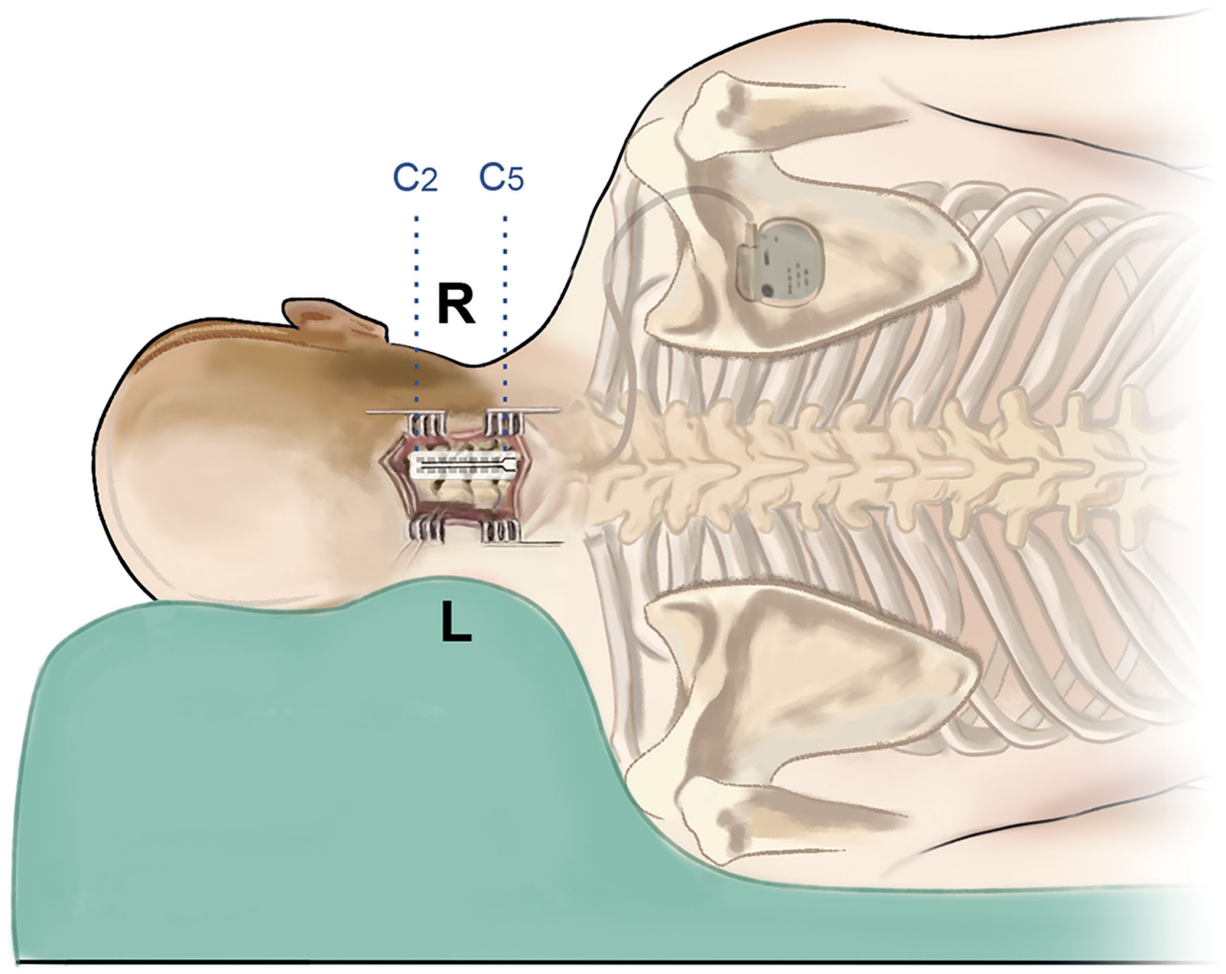
Schematic image of the surgical procedure. The patient is in the left lateral decubitus position, and the incision is at the C2–C5 level. After implantation of the double-paddle electrodes, the stimulator was fixed at the chest wall.

In this study, all the surgeries were performed by the same senior physician (J.H.), and he is right-handed. The patient was under general anesthesia and placed in a lateral decubitus position (Figure 2). The surgical approach is the posterior median approach, and the incision was made at the level of C2–C5, generally centering on the spinous process of C5. After the incision, the muscles were separated to the lamina, and C5 was bitten off with a part of the lamina and ligamentum flavum removed. Part of C3 was also bitten off which provided an observation window to prevent the deviation of the electrode. Surgical stimulation electrodes (39286, Medtronic Inc., Minneapolis, MN) were implanted into the epidural cervical spinal canal at the level of C2–C5 through the epidural space. After the electrodes were implanted in place, we utilized tight sutures for anchoring and sealing the bone window with bone wax. Immediately after the operation, the location of the electrode was confirmed by C-arm. A pulse generator (Medtronic, USA) was placed under the anterior chest wall, and the electrodes were connected to the pulse generator through a subcutaneous tunnel.

### Postoperative Stimulator Settings

One week after the operation, the pulse generator was turned on after the patient's condition was stable. The pulse width was set to 210 us, the frequency was 5 Hz, and different voltages (0.5, −2, and 0.5 V increments each time) were used for testing. The occurrence of limb tremor due to stimulation was observed, and the voltage at the previous level of the tremor was decided as the stimulation voltage.

### Statistical Analysis

SPSS 20.0 software was used for data statistics and analysis. Shapiro–Wilk test was used to judge the normality of continuous variables. Normally distributed or non-normally distributed continuous variables are presented as mean ± standard deviation or median (IQR), respectively. Data was tested for homogeneity of variance. According to the variance, Chi-square test or two-way repeated-measures analysis was used. *P* < 0.05 was considered to be significantly different.

## Results

### Preoperative Factors on SCS Electrode Deviation

Among patients who received SCS treatment in our institutions from 1 January 2010 to 31 December 2020, 27 patients were found to have electrode deviation postoperatively and enrolled in the analysis. The mean age was 48.48 ± 13.98 years. About 70.4% of the patients were male (Table 1). Concerning the pathogeny, DOC caused by stroke in the more variation group (*n* = 3) was less than those in the less deviation group (n = 9), although no significant difference was found. The median course of DOC was 210 (158–323) days, with no significant difference between the two groups. We also studied the C2–C5 distance and found an average of 69.5 ± 5.1 mm. The spinal cord to spinal canal ratio was studied with an average of 0.36 ± 0.05, and no significant difference was found between the groups. Preoperative consciousness evaluation was also performed using JFK CRS-R, and the scores in the more deviation group were less than the scores in the less deviation group, and there was no significant difference.

**Table 1:** Preoperative factors on SCS electrode deviation.

**Variables**	**Total** **(*N* = 27)**	**More deviation group** **(*N* = 12)**	**Less deviation group** **(*N* = 15)**	***p*** **-value**
Sex, male (%)	19 (70.4)	7 (58.3)	12 (80.0)	0.22
Age, y (mean ± SD)	48.48 ± 13.98	46.08 ± 15.72	50.4 ± 12.65	0.44
Pathogeny, *n* (%)				0.15
Stroke	12 (44.4)	3 (25.0)	9 (60.0)	
Trauma	11 (40.7)	6 (50.0)	5 (33.3)	
Anoxia	4 (14.8)	3 (25.0)	1 (6.7)	
Course, d (median, IQR)	210 (158–323)	209 (169–417)	215 (132–323)	0.79
C2–C5 distance, mm (mean ± SD)	69.5 ± 5.1	68.1 ± 6.0	70.7 ± 4.1	0.21
C2 spinal cord to spinal canal ratio (mean ± SD)	0.36 ± 0.05	0.36 ± 0.05	0.34 ± 0.05	0.50
Preoperative CRS-R, point (mean ± SD)	6.96 ± 1.79	6.75 ± 2.05	7.13 ± 1.60	0.59

*Variables are represented as mean ± standard deviation or median (IQR)*.

* *P < 0.05, significant difference*.

### The Influence of Surgical Position on Electrode Deviation

We explored whether the surgical position would affect the direction and angle when electrode deviation occurred in the patients. The results showed that the incidence of left lateral decubitus position in patients with right deviations of electrodes was 64.7% (11 / 17), and the incidence of right lateral decubitus in patients with left deviations of electrodes was 80% (8/10) (Table 2). The association between surgical position and deviation direction was significantly different (*P* = 0.025).

**Table 2:** Electrode position and surgical position relationship.

**Electrode deviation direction**	**Left lateral decubitus**	**Right lateral decubitus**	***X*** **^2^**	***p*** **-value**
Right	11	6		
Left	2	8	5.0405	0.025

* *P < 0.05, significant difference*.

The angles of electrode deviation were also explored between the groups. In 27 patients, the maximum angle of the first electrode deviation was 54° and the minimum was 1°. The average deviation was 36°. The average deviation angles in the less deviation group (14.6, 10.47, and 4.47° at the 1st, 2nd, and 3rd electrodes) were significantly lower than the angles in the more deviation group (41.08, 30.92, and 24.00°) (*P* < 0.001). C2–C5 axial deviation angle gradually decreases in both the groups.

### Therapeutic Effects and Complications According to SCS Electrode Deviation

We further explored whether therapeutic effects and complications differ according to SCS electrode deviation. The results showed that the stimulation intensity in the less deviation group (1.70 ± 0.41) was significantly higher than in the more deviation group (1.25 ± 0.34) (*P* = 0.006) (Table 3).

**Table 3:** Side effects according to SCS electrode deviation.

	**Total (*N* = 27)**	**More deviation group** **(*N* = 12)**	**Less deviation group** **(*N* = 15)**	***p*** **-value**
**Electrode deviation direction**, ***n*** **(%)**				0.722
Left	10 (37.0)	4 (33.3)	6 (40.0)	
Right	17 (63.0)	8 (66.7)	9 (60.0)	
1st EDA, degree (mean ± SD)	26.37 ± 15.43	41.08 ± 6.89	14.6 ± 8.42	<0.001
2nd EDA, degree (mean ± SD)	19.56 ± 13.04	30.92 ± 8.50	10.47 ± 7.73	<0.001
3rd EDA, degree (mean ± SD)	13.15 ± 13.36	24.00 ± 11.72	4.47 ± 6.46	<0.001
Stimulation intensity, V (mean ± SD)	1.50 ± 0.44	1.25 ± 0.34	1.70 ± 0.41	0.006
Limb tremor*, ***n*** **(%)**				
Left	4 (14.8)	3 (25.0)	1 (6.7)	
Right	7 (25.9)	6 (50.0)	1 (6.7)	0.049
Bilateral	16 (59.3)	3 (25.0)	13 (86.6)	
PSH episodes, *n* (%)	6 (22.2)	5 (41.7)	1 (6.7)	0.030

* *Limb tremor was assessed at the strongest stimulation intensity*.

*Variables are represented as mean ± standard deviation*.

*P < 0.05, significant difference*.

Under the strongest stimulation voltage at 5 Hz in each group, the tremor of the upper limbs on the deviation side of the more deviation group was more obvious than that of the less deviation group, and the tremor of bilateral upper limbs of the less deviation group was more obvious (*P* = 0.049). As for complications, we found more PSH episodes in the more deviation group (*n* = 5) than in the less deviation group (*n* = 1), which was significantly different (*P* = 0.030).

**Figure 3:**
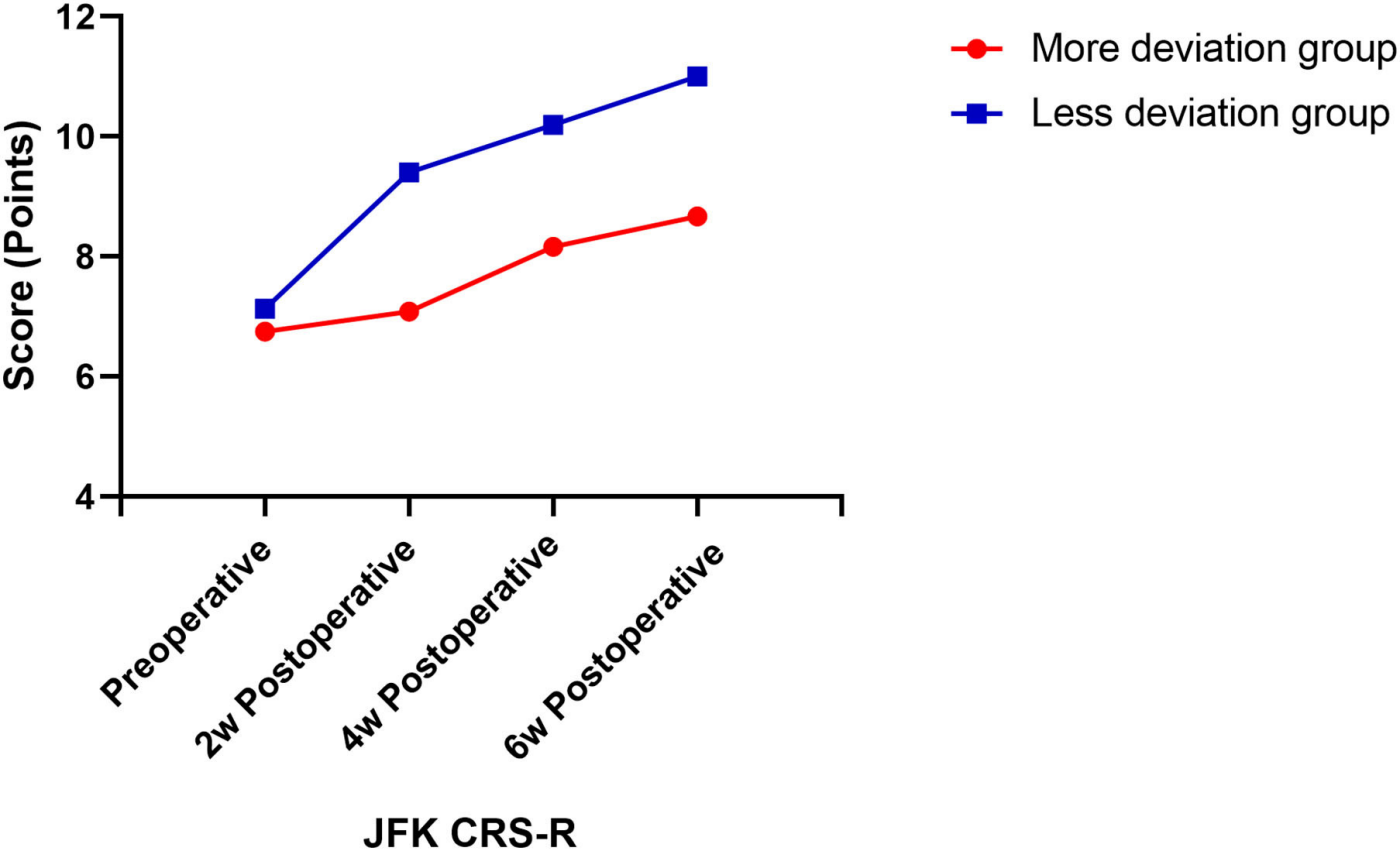
Postoperative CRS-R score change curve. CRS-R scores were assessed at four time-points, which were preoperative, 2 weeks, 4 weeks, and 6 weeks postoperatively.

Then, we assessed the patients' consciousness levels using JFK CRS-R at 2, 4, and 6 weeks postoperatively. The results showed EDA significantly affects the postoperative CRS-R of patients with DOC, and patients in the less deviation group had significantly better postoperative CRS-R (*P* < 0.01) (Table 4). With the prolonged postoperative recovery time of 6 weeks, CRS-R gradually increased (*P* < 0.05). There was also an interaction effect between EDA and postoperative time. With the prolonged postoperative time, the CRS-R improvement rate of patients with different EDA was different, and the CRS-R improved faster in patients with less EDA (*P* < 0.05; Figure 3).

**Table 4:** The effect of EDA on treatment effect over time.

**Time point**	**More deviation group (*N* = 12)**	**Less deviation group (*N* = 15)**	**F**			
			**Time**	**Group**	**Time × Group**	**Es (η^2^)**
Preoperative CRS-R, point (mean ± SD)	6.75 ± 2.05	7.13 ± 1.60	39.406**	5.236*	3.255*	0.298
Postoperative CRS-R at week 2, point (mean ± SD)	7.08 ± 2.07	9.40 ± 2.72				
Postoperative CRS-R at week 4, point (mean ± SD)	8.16 ± 1.80	10.20 ± 2.31				
Postoperative CRS-R at week 6, point (mean ± SD)	8.67 ± 1.81	11.00 ± 2.39				

*Variables are represented as mean ± standard deviation*.

* *P <0.05*,

** *P < 0.01*.

## Discussion

In this study, we demonstrated that in patients with DOC who received SCS treatment, surgical positions are significantly related to the electrode deviation direction. No significant difference was found in age, sex, duration of DOC, and other baseline characteristics. Patients with more EDA are less likely to be tolerant to stimulation intensities and thus more prone to complications such as upper limb tremors and PSH. We also found that SCS treatment can improve the outcome of patients, and the therapeutic effects measured by JFK CRS-R for patients after surgery were all improved compared with preoperative evaluation. The CRS-R scores in the more deviation group were worse than that in the less deviation group, and the CRS-R improved slower in patients with more EDA. The above results suggest the vital role of EDA in the outcome of SCS treatment.

With the advancement of medical technology, the survival rate of critically ill patients has increased significantly. Though they survived, many of them transitioned from coma states to chronic DOC (coma > 28d) (17). In the research field of DOC, the current main treatment methods include drug therapy, non-invasive neuromodulation, and invasive neuromodulation (18). While amantadine has been verified in clinical randomized controlled trials (RCTs), most of the drugs and surgeries currently still lack evidence that they can improve the patients' prognosis (19, 20). After more than 50 years of development, deep brain stimulation (DBS), spinal cord stimulation, and vagus nerve stimulation are more commonly used in invasive neuromodulation therapy (2, 21, 22). Spinal cord stimulation treatment, as an invasive neuromodulation technique, is increasingly used in the treatment of DOC (23, 24). As for the underlying mechanisms, studies have shown that after receiving SCS treatment, the cerebral blood flow (CBF) was significantly increased (13, 25, 26). There is also more evidence proving the effect of SCS was through promoting the cortical pathway, stimulating reticulo–thalamo–cortical pathway, and improving functional activity in the brain circuits that are the neuronal substrates of DOC (7, 8, 11, 27). Since 1988, there are 318 patients with DOC who are reported to be treated by the SCS treatment with a total effective rate of 52.2%.

In our study, we recruited patients with a duration of DOC >3 months and no signs of spontaneous improvement within 8 weeks. The results showed significant improvement in patients receiving SCS implantation. However, the related factors that affect the therapeutic effect are mainly studied from the aspect of preoperative screening of patients, and there is no systematic study on intraoperative position and postoperative electrode position. During the surgical procedure, some patients' single-column paddle may not completely cover the dorsal column. For patients receiving multi-column paddle operations, the deviation of the electrode may result in only one contact in a pair that covers the midline. The ideal electrode position is the guarantee of treatment effect. Thus, we propose to explore the related factors on postoperative EDA, complications, and therapeutic effects.

After SCS implantation, the impulses generated by stimulation will produce retrograde impulses and anterograde pain nerve impulses. Retrograde impulses mainly activate the reticular ascending system, which promotes emerging. Anterograde impulses stimulate spinal motor neurons, which in turn increase the excitability of spinal motor neurons, causing the corresponding muscles (corresponding upper limbs) to contract. For patients with DOC, the greater the stimulation intensity, the better the stimulation of the reticular ascending system (28). But tremors in the corresponding upper limbs with increased stimulation can be seen as a complication and may cause discomfort to the patient. When the EDA is >30°, the stimulation to the motor neurons of the spinal cord on the deviation side is significantly enhanced, which is manifested as contraction tremors of the muscles of the limbs on the same side. As a result, stimulation intensity was significantly reduced in this group compared to the less deviation group, which in turn reduced the therapeutic effect. PSH also hinders the restoration of consciousness and often occurs in patients with extensive neurological damage, where damage to the brainstem and diencephalon relieves excitatory spinal processes from their inhibitory effects (29). However, most patients with DOC have extensive neurological damage and are prone to induce PSH. At present, there are very few PSH-related studies in patients with DOC. Pignolo et al. studied patients with vegetative states and showed that the incidence of PSH was 26.1% (30). There is still no standardized treatment plan for PSH in DOC patients. In the course of treatment, symptomatic treatment or avoidance of inducing factors are mainly adopted, and symptoms are mainly controlled by heart rate control (non-selective beta-blockers) and sedation (benzodiazepines). However, the description of sedative drugs in patients with DOC is detrimental to recovery. Our study found that patients with greater EDA were more likely to induce PSH episodes in patients. The symptoms of PSH episodes in patients with DOC are mainly increased heart rate and blood pressure, and some patients can be relieved after being given an appropriate dose of painkillers. The symptoms in these cases are mild, and in some patients, they may also be accompanied by painful expressions. Considering the difference between PSH episodes and primary brain damage, it is more likely to be related to the unequal stimulation intensity on both sides caused by the increase in the EDA, which increases the patient's discomfort and induces PSH. Therefore, reducing the EDA during SCS surgical procedures can effectively reduce the occurrence of this situation and improve the patients' outcomes.

In SCS treatment of patients with DOC, attention should be paid to the influence of surgical position on the direction of electrode deviation intraoperatively. Our team has tried surgery with patients in the prone position, but there is no difference in the EDA from the lateral position. Also, auxiliary methods such as intraoperative C-arm/intraoperative electrophysiology should be combined to minimize the EDA. The surgical procedure also has the potential to be improved to reduce the electrode excursion probability, which may be one of the future research directions. We utilized tight sutures for anchoring and sealing the bone window with bone wax, all of which helped to minimize unnecessary EDA. Considering that DOC patients do not have spontaneous vigorous activity, vigorous shaking should also be reduced during postoperative care. Once the EDA is reduced, the maximum stimulation intensity can be increased, and then the patient's CRS-R score is improved and the chance of inducing PSH is lowered, thereby improving the patient's prognosis.

There are still limitations in this study. This study is a retrospective cohort, and there is a potential bias. Second, the small sample size of this study makes it difficult for us to fit linear models of EDA and functional prognosis, which might be of interest. Still, we bit off part a of C3, which provides an observation window to prevent the electrode from deviation. The surgery can further be measured by neurophysiological methods and the implantation process can be carried out under the C-arm in the future. Better surgical improvement solution to reduce EDA is still needed, which might be discussed in our future research. Finally, considering that this article is a retrospective study, there are no blank controls in our study. However, spinal cord stimulation has been proven to be effective in the treatment of DOC. In this article, we mainly study the factors that affect the efficacy, which is sufficient in clarifying the issues. Therefore, large-scale prospective RCTs are still needed to further explore this issue.

## Conclusion

We found that electrode deviation will affect the outcome of patients receiving cervical SCS treatment, and the intraoperative surgical position is associated with postoperative electrode deviation direction. The reduction of EDA under 30° can increase maximum tolerant stimulation intensity, reduce complications, and further improve patients' outcomes. Modification of surgical procedures to reduce EDA and large-scaled clinical studies should be carried out to further explore this question.

## Data Availability Statement

The raw data supporting the conclusions of this article will be made available by the authors, without undue reservation.

## Ethics Statement

The studies involving human participants were reviewed and approved by Ethics Committee of Beijing Tiantan Hospital, Capital Medical University (KYSQ 2020-387-01). The patients/participants provided their written informed consent to participate in this study.

## Author Contributions

QH wrote the manuscript. BH, XX, YD, and XC collected data. JH and YY supervised the study and reviewed the manuscript. All authors contributed to the article and approved the submitted version.

## Funding

This research was supported by the following funding sources: National Natural Science Foundation of China (No. 81600919). Beijing Municipal Science and Technology Commission (Nos. Z161100000516165 and Z171100001017162). Beijing Nova Program (Z181100006218050).

## Conflict of Interest

The authors declare that the research was conducted in the absence of any commercial or financial relationships that could be construed as a potential conflict of interest.

## Publisher's Note

All claims expressed in this article are solely those of the authors and do not necessarily represent those of their affiliated organizations, or those of the publisher, the editors and the reviewers. Any product that may be evaluated in this article, or claim that may be made by its manufacturer, is not guaranteed or endorsed by the publisher.
